# Alizarin Red S for Online Pyrophosphate Detection Identified by a Rapid Screening Method

**DOI:** 10.1038/srep45085

**Published:** 2017-03-24

**Authors:** Jens Fischbach, Qiuting Loh, Frank F. Bier, Theam Soon Lim, Marcus Frohme, Jörn Glökler

**Affiliations:** 1 Division Molecular Biotechnology and Functional Genomics, Technical University of Applied Sciences Wildau, Hochschulring 1, 15745 Wildau, Germany; 2 Institute for Research in Molecular Medicine, Universiti Sains Malaysia, 11800 Penang, Malaysia; 3 Institute for Biochemistry and Biology, University of Potsdam, Karl-Liebknecht-Straße 24-25, 14476 Potsdam, Germany; 4 Analytical Biochemistry Research Centre, Universiti Sains Malaysia, 11800 Penang, Malaysia

## Abstract

We identified Alizarin Red S and other well known fluorescent dyes useful for the online detection of pyrophosphate in enzymatic assays, including the loop mediated isothermal amplification (LAMP) and polymerase chain reaction (PCR) assays. An iterative screening was used for a selected set of compounds to first secure enzyme compatibility, evaluate inorganic pyrophosphate sensitivity in the presence of manganese as quencher and optimize conditions for an online detection. Of the selected dyes, the inexpensive alizarin red S was found to selectively detect pyrophosphate under LAMP and PCR conditions and is superior with respect to its defined red-shifted spectrum, long shelf life and low toxicity. In addition, the newly identified properties may also be useful in other enzymatic assays which do not generate nucleic acids but are based on inorganic pyrophosphate. Finally, we propose that our screening method may provide a blueprint for rapid screening of compounds for detecting inorganic pyrophosphate.

Hydrolysis of nucleotide triphosphates and the release of inorganic pyrophosphate (PPi) is of central importance in cellular metabolism as it serves as a committed step in anabolic synthesis due to the concomitant hydrolysis of pyrophosphate. The *in vitro* monitoring of reactions such as hydrolysis of adenosine triphosphate (ATP)^1^, generation of cyclic adenosine monophosphate (cAMP)^2^, acetyl-coenzmye A catalyzed activation of fatty acids^3^, RNA transcription and replication of DNA^4^,^5^ is generally carried out by both enzymatic and enzyme-free assays. Many of the enzymatic assays are based on multi-component reactions coupled with UV-vis spectrophotometric, luminescent or fluorescent detection^6^,^7^,^8^,^9^,^10^. Due to their high sensitivity, enzyme-based assays are applicable to clinical diagnostics and research, but are otherwise time-consuming, have limited shelf-life, and are cost-intensive. New sensory receptor molecules (chemosensors) transducing chemical into optical signals are used to overcome some of these disadvantages^11^. However, despite their high selectivity and sensitivity, many chemosensors are based on non-commercial chemical derivatives and thus less accessible for quick adoption or applications and investigations in basic research^12^,^13^,^14^.

PPi forms complexes with multivalent cations, thus it can be used in assays based on fluorescent dyes that are initially quenched by Mn^2+^, Cu^2+^, Co^2+^ or Ni^2+^ ions. These metal ions are efficiently scavenged by PPi resulting in an increased fluorescent signal for detection^15^. This quencher displacement approach (turn-ON assay) is exploited for metal ion detection in titration experiments or monitoring biochemical reactions with complex-forming fluorescent dyes such as calcein, having strong affinity for Mg^2+^ and Ca^2+^ ions^16^. These dyes are mainly used for endpoint detection and have a defined but also narrow absorption- and emission maxima that may limit their applicability. Excess PPi accumulation leads to an increased turbidity which can be used to indirectly detect DNA polymerisation during loop-mediated isothermal amplification (LAMP)^17^,^18^. A direct fluorescence-based approach has been developed with manganese-quenched calcein that becomes strongly fluorescent when manganese ions are sequestered by newly generated PPi^19^.

Our goal was to identify inexpensive, commercially available dyes with new properties for online PPi detection in enzymatic reactions. As we have discovered previously that metal ions such as Zn^2+^ are not as selective for pyrophosphate as Mn^2+^, especially with respect to nucleotide triphosphates that are present in the reactions (data not shown), we have focused on screening for Mn^2+^-dependent dyes alone (see Fig. 1). As a starting point, we investigated the PPi-dependent quenching properties of Mn^2+^ for several dyes that are known to bind Ca^2+^, and/or Mn^2+^. Based on their good availability we selected, calcein blue (CAB), folic acid (FOL), tetracycline (TET), doxycycline (DOX), alizarin red S (ARS), and calcein (CAL) as a positive control. CAL^20^ as well as CAB^21^ are used for *in vitro* viability studies and in histochemistry. FOL can be synthesized in large scale and is involved in carbon transfer reactions *in vivo*^22^. The two antibiotics TET, a natural dye, and DOX, a synthetic derivative as well as the synthetic dye ARS are typically used for bone staining^23^,^24^,^25^. The most crucial test for identification of useful PPi chemosensors is a test for any inhibitory properties and/or tolerated concentration range with regard to the respective enzyme. In addition, the evaluation of fluorescent dyes for sensitive online detection purposes may require time-consuming and expensive validation of the assay under real enzymatic reaction conditions. Thus, we have devised an initial combinatorial screening assay mimicking the required reaction conditions.

## Results

### Effect of manganese and magnesium on selected fluorescence dyes under LAMP and PCR conditions

Before the effect of the metal ions can be addressed, one has to consider the selectivity of Mn^2 +^ to PPi in presence of dNTPs (Supplementary Fig. S1). The effect was found to be minor in the presence of magnesium and therefore dNTPs were omitted in the first experiments.

The quencher-displacement assay for detecting PPi using metal-dependent fluorescent dyes was preliminarily investigated by fluorescence intensity measurements of conventional dyes in the absence or presence of Mg^2+^ and Mn^2+^ under typical LAMP reaction conditions. All tested dyes resulted in varying fluorescence decrease in the presence of Mn^2+^ (Fig. 2). The strongest response to manganese ions is achieved by CAL and ARS (Fig. 2a and e). The data also indicates that both metal-ions strongly influence fluorescence quenching of all dyes except for ARS which also exhibits a strong fluorescence increase in the presence of Mg^2+^ alone (Fig. 2e). The quenching effect by Mn^2+^ was also confirmed under PCR conditions. Based on previous investigations, further tests under simulated reaction conditions of LAMP and PCR revealed different signal-to-noise ratios (SNR), that was calculated between samples in presence and absence of PPi for all dyes (see supplementary information). The simulation mimics the enzymatic amplification of DNA with accumulating PPi. The highest SNR is achieved with CAL (8-fold), TET (5-fold) and ARS (4-fold) under LAMP conditions. The corresponding values for PCR are lower (Supplementary Fig. S2).

### Effect of Mn^2+^, tetracycline and alizarin red S on the Bst- and Taq DNA polymerase

Mg^2+^ is a basic requirement for the polymerisation reaction while Mn^2+^ often results in inhibition at higher concentrations. Thus, we tested both amplification reactions with increasing Mn^2+^ concentrations up to 1 mM in combination with increasing concentrations of TET and ARS which revealed the highest SNR values among all tested dyes and have not been used in LAMP and PCR. The gel electrophoretic analysis of the PCR and LAMP products indicates that Mn^2+^ inhibits the reaction of the Taq DNA polymerase at 1 mM (Fig. 3a). The typical ladder-like pattern observed in LAMP reactions indicate a slight intensity decrease with increasing Mn^2+^ concentration but the reaction itself was not inhibited completely as amplified DNA was present in all samples (Fig. 3b). TET does not reduce the amplification efficiency of neither Taq nor Bst DNA polymerase. ARS inhibits the Taq DNA polymerase above 0.1 mM, but not the Bst DNA polymerase.

### Combinatorial screening assay

In order to save enzyme and oligonucleotide expenses, a combinatorial screening under simulated assay conditions with anticipated PPi endpoint concentrations was performed. The simulated screening was limited to conditions that are compatible with the enzymatic assays. As the selected dyes (TET, ARS and CAL as control) are quenched by Mn^2+^, combinations with dye concentrations exceeding the Mn^2+^ concentrations were omitted. The final signal-to-noise ratio was calculated by measuring the fluorescence intensities of each combination in the microtiter plate-based assay with and without PPi under simulated reaction conditions of both LAMP and PCR. The resulting fluorescence intensities of each measurement were analysed and displayed as heatmap (Figs 4 and 5).

The highest SNR value for LAMP conditions was achieved with 25 to 50 μM CAL and 0.5 mM Mn^2+^ (Fig. 4a). As the heatmap indicates, nearly all SNR values with given concentrations of CAL and Mn^2+^ range between 4 and 13 except for the highest concentration of Mn^2+^ (Fig. 4a). The respective control with selected combinations in a real LAMP-assay (Fig. 4a bottom) revealed a SNR value around 2 to 5, which is significantly less than the simulated value. This discrepancy was more pronounced for CAL when compared to the other dyes. The SNR values of TET under LAMP conditions are between 1.1 and 1.5 (Fig. 4b) with the highest ratio for 100 to 250 μM and 0.5 to 1 mM Mn^2+^. The heatmap also indicates a correlation between the SNR and Mg^2+^ concentration as it decreases with increasing amount of Mg^2+^. The respective SNR values of the real LAMP reaction confirmed some of the combinations. ARS revealed SNR values between 2 and 7 in a range of 50 to 250 μM with 0.5 to 1 mM Mn^2+^ (Fig. 4c). This range could be confirmed by testing selected combinations in a real LAMP reaction resulting in lower SNR values than expected from the simulation (Fig. 4c bottom).

The screening assay under simulated PCR reaction conditions was adjusted to a PPi concentration of 0.25 mM as the amount of generated PPi is much less than in LAMP reactions. Compared to the LAMP data, the SNR value of CAL in PCR was 10-fold lower (Fig. 5a). The real PCR reaction revealed SNR values of 1.6 to 1.8 for two tested combinations. TET worked best with 250 μM and 0.25 mM Mn^2+^ under PCR conditions (Fig. 5b). The comparison with real reaction data revealed SNR values of 1.4. In PCR, only the lowest concentration of ARS and 0.1 mM Mn^2+^ revealed a SNR value of 1.8 (Fig. 5c). To show the applicability of the screening we also performed the screening with FOL, DOX and CAB under simulated LAMP conditions.

### Detection range of inorganic pyrophosphate for selected dyes

The determined optimal combinations (Supplemental Table 3) were finally used to test the correlation between fluorescent signal and increasing PPi. The dynamic range for detecting PPi with all three dyes under LAMP and PCR conditions was investigated for CAL, TET and ARS. The fluorescent signal was detected in a range of 0 to 2 mM PPi for LAMP and 0 to 0.5 mM in PCR conditions (see Fig. 6). The fluorescence intensity of all three dyes increased in the range of up to 1.25 mM PPi under LAMP conditions and up to 0.5 mM when PCR conditions are applied. In contrast to CAL and ARS, the initial fluorescence intensity without any PPi of TET was considerably higher.

## Discussion

The detection of PPi by the proposed “turn-On” assay is based on the efficient and specific displacement of a metal ion from a fluorescent dye by PPi. The divalent Mn^2+^ ions are known to strongly bind to PPi and have a comparably low affinity towards other polycations such as dNTP. Most other polyvalent metal ions are also not sufficiently selective for PPi and/or require conditions and buffers not compatible with polymerase reactions. In the presence of excess Mg^2+^, most of the Mn^2+^ is bound to PPi due to a much higher affinity. Thus, Mg^2+^ can be seen as a highly effective blocking reagent that allows selective binding of Mn^2+^ to PPi even in the presence of elevated concentrations of dNTP. Therefore, the inexpensive Mg^2+^ was varied in the screening, whereas the more expensive dNTPs were not included.

The initial quenching is based on coordination of paramagnetic metal ions such as Mn^2+^ 26. All tested dyes show a cation-induced fluorescence reduction in presence of Mn^2+^. The levels of the fluorescence decrease under primarily selected reaction conditions for LAMP and PCR differ among the investigated dyes. Other metal-ions such as Mg^2+^ are essential for the activity of most DNA polymerases and known to enhance the fluorescence response. This counteracts the Mn^2+^-dependent quenching of the dye. Thus, the amount of both cations must be adjusted to achieve a high signal-to-noise ratio (Supplementary Fig. S3). Therefore, CAL, TET and ARS were selected for further screening among all tested dyes.

The presence and concentration of divalent metal ions not only influence the fluorescence of a dye, but also the reaction efficiency of the enzymatic reactions as well. DNA polymerases such as the Taq and Bst DNA polymerases require a certain level of divalent metal-ions as cofactor for optimal performance^27^. Inhibitory effects on the enzymatic reaction by Mn^2+^ and the selected dyes have to be checked first. TET can be used in both reactions even at high concentrations while ARS above 0.1 mM concentrations can be applied in LAMP. Higher concentrations of ARS may bind to much Mg^2+^ which lowers the overall activity of the Taq DNA polymerase. The Bst DNA Polymerase can compensate for higher amounts of ARS due to the higher concentration of Mg^2+^ in the LAMP reaction. High Mn^2+^ concentrations may increase base misincorporation during the polymerization reaction and thus decrease the fidelity of the polymerase^28^.

The spectral characterization of the dyes allows the use of more appropriate filter sets and may result in a reduced background. In contrast to calcein (Ex_max_ = 495 nm/Em_max_ = 520 nm), the distance between maximal excitation and emission wavelength is greater for tetracycline (Ex_max_ = 370 nm/Em_max_ = 530 nm) and alizarin red S (Ex_max_ = 535 nm/Em_max_ = 645 nm). The peaks of the excitation and emission spectra of TET and ARS are set apart by more than 150 nm compared to CAL with just 75 nm. Thus, calcein has a narrow window for excitation and emission spectra which restricts the measurement to more distinct filter settings (Supplementary Fig. S4).

To simplify the adjustment of all relevant components, microtiter plate screening under simulated conditions allows testing of all combinations in parallel. To keep the consumption of enzyme and oligonucleotides and dNTPs low, the screening was limited to metal ions that contribute to the detection signal. However, if the enzymatic assay costs are not limiting, it is well possible to skip the simulated assay to directly arrive at optimal conditions and best performance. In addition, a heatmap diagram is a simple and effective way to quickly analyze the complex experimental data for optimal combinations. The screening with FOL, DOX and CAB did result in poor SNR values and therefore, these dyes were not considered in the following experiments (Supplemental Fig. S5).

The difference of the signal-to-noise ratio of CAL and ARS between the simulated screening and real enzymatic assays of LAMP and PCR can be attributed to the complex interactions between Mg^2+^ and Mn^2+^ binding components that are present in the enzymatic reaction. Oligonucleotides are known bind Mg^2+^ and Mn^2+^ as well as deoxynucleotide triphosphates (dNTPs), but less obvious may be interference by additional interactions with the dyes themselves. The enzyme that catalyses the polymerization reaction also binds divalent metal ions as cofactor at a much lower concentration than the aforementioned compounds. Dependent on the input amount of dNTPs and the reaction efficiency, the amount of liberated inorganic PPi varies strongly. Assuming that a LAMP reaction yields up to 20 μg DNA in a 25 μL reaction and one PPi molecule is generated per incorporated nucleotide, 1 to 2 mM of PPi will be generated in LAMP^20^. Depending on the efficiency of the PCR reaction, the initial primer, template and dNTP concentration, less than 0.5 mM of PPi will be generated in PCR. In the endpoint both LAMP and PCR will yield template-length independent amounts of PPi.

Three dyes could be identified to allow the detection of inorganic pyrophosphate derived from LAMP and PCR. For both reactions the detectable range of PPi is covered by the fluorescent measurements with all three dyes. In contrast to TET which needs further optimisation due to higher background signal, CAL and ARS performed well under LAMP and PCR conditions. The low signal-to-noise ratio of TET can be explained by a lower affinity towards Mn^2+^. To simplify and accelerate the use of other low-cost and accessible dyes for pyrophosphate detection in enzymatic reactions, we propose an iterative workflow outlined in Fig. 7.

At the beginning it is recommended to limit the number of dyes that change spectral properties in the presence of divalent metal ions by to complex formation with PPi (1). A prescreening with selected dyes under conditions that are similar to the specific enzymatic reaction restricts the number of dyes that exhibit the desired effect (such as fluorescence quenching) (2). Exclusion of inhibitory effects of dyes and metal ions on the enzyme(s) should be investigated directly with varying concentrations (3). To optimize the signal response in the presence of PPi it is recommended to carry out a combinatorial screening in a microtiter plate based assay. Depending on the throughput of the screening with respect to number of parallel assays, total volumes and overall costs of the enzymatic reaction, the screening may be performed under simulated (4a) or real enzymatic conditions (4b). The latter does not require additional verification. The resulting multiparametric fluorescence data can be very simply interpreted based on heatmaps (Fig. 5a and b). Finally, the verification of the optimal combinations is carried out in an enzymatic reaction if screening was performed under simulated conditions (6).

## Conclusion

In conclusion, we established a rapid and simple method for fluorescence based PPi detection using a microtiter plate assay mimicking endpoint conditions of both LAMP and PCR. The obtained data was analysed by calculating the SNR and heatmaps. Additional enzymatic assays were performed to confirm the identified working range. We demonstrated that the conventional and well studied dyes alizarin red S, and to a lesser extent tetracycline, can be repurposed for detection of PPi in a quencher-displacement assay format based on highly selective manganese ions. Furthermore, the dyes allow online detection of PPi in applications that require different spectra than calcein. Especially, the very inexpensive dye alizarin red S was found to be very useful due to its sensitivity, defined red-shifted spectrum, long shelf life and low toxicity. The screening can be adapted to all types of enzymatic reactions that either generate or consume PPi and is not limited to nucleic acid amplification reactions. Based on our results, we expect that many other dyes, especially those of known binding to alkaline earth metal ions or staining mineralized tissues comprising phosphate can be identified by our screening method and put to a novel use for the online detection of PPi in enzymatic reactions.

## Methods

### Combinatorial screening assay

**Figure 1:**
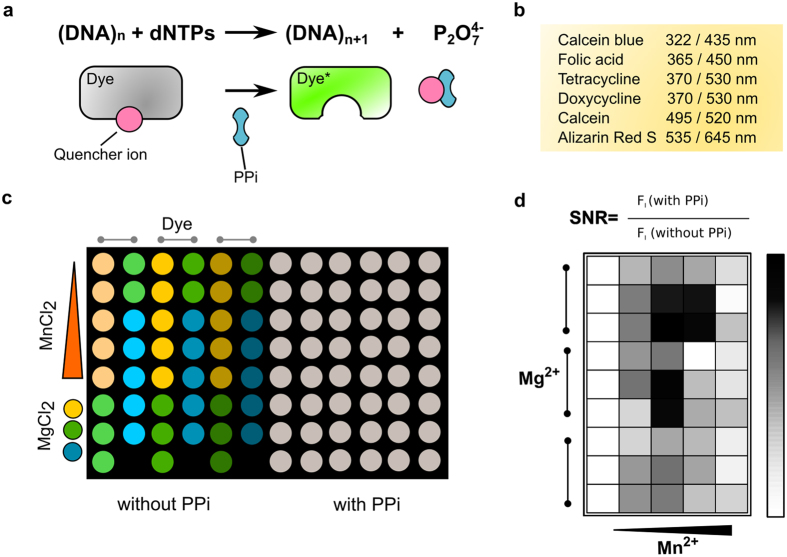
Scheme of displacement assay and screening. (**a**) Quencher (pink) displacement by accumulating PPi (blue) with fluorescent response of the dye (green). (**b**) Selected dyes with respective excitation and emission maxima. (**c**) Microtiter plate screening layout comprising three dye concentrations, three Mg^2+^ and five Mn^2+^ (quencher) concentrations. Each well represents one combination of all components with or without PPi to mimic the endpoint of a biochemical reaction. (**d**) The ratio of positive (accumulating PPi) and negative (no PPi) fluorescent values is given as signal-to-noise ratio (SNR) and visualized as heatmap.

**Figure 2:**
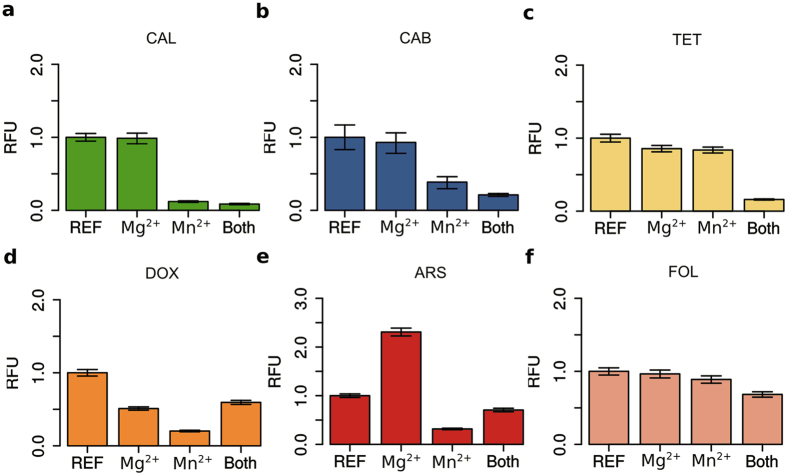
Prescreening of conventional dyes with response to Mg^2+^ and/or Mn^2+^ under LAMP conditions. Fluorescence measurements were carried out with 25 μM calcein (**a**), calcein blue (**b**), tetracycline (**c**), doxycycline (**d**), alizarin red S (**e**), folic acid (f) in 50 mM Tris/Hcl buffer (pH 8.8). with supplemented Mg^2+^ (7 mM), Mn^2+^ (0.5 mM) as well as a combination of both. The values were normalized to the reference representing the dye only. Values above 1.0 correspond to an increase and below to fluorescence quenching. The samples were prepared in triplicates and represented by error bars.

**Figure 3:**
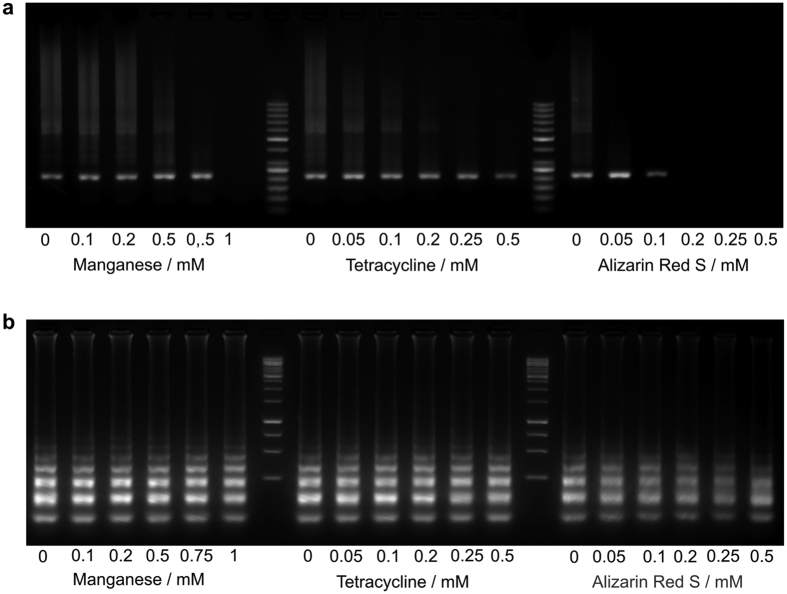
Effect of manganese, tetracycline and alizarin red S on the DNA polymerases. Manganesechloride, tetracycline and alizarin red S were used to investigate the effect on the Taq DNA polymerase (**a**) and Bst DNA polymerase (**b**) under real amplification reaction conditions. Gel electrophoresis of all PCR and LAMP samples with increasing concentrations from left to right.

**Figure 4:**
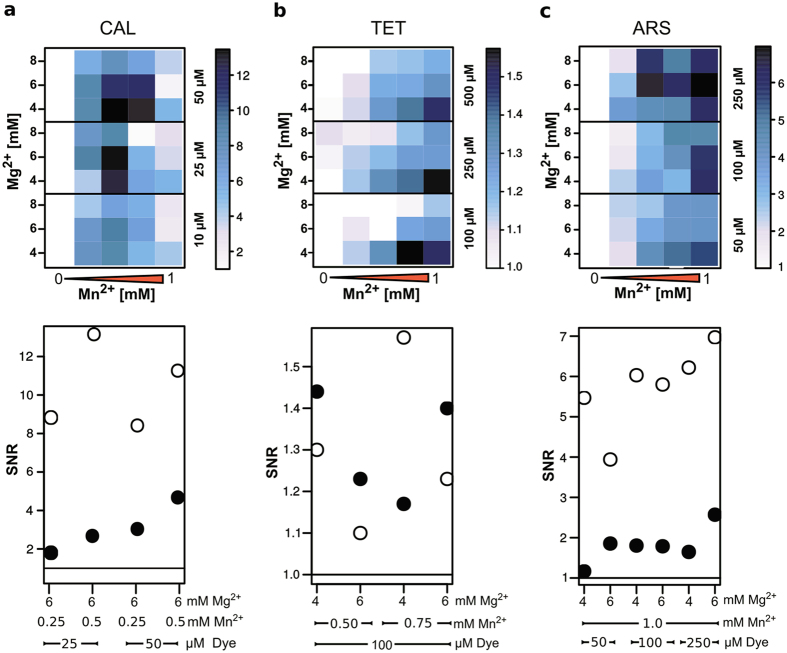
Heatmap analysis of signal-to-noise ratio (SNR) of simulated LAMP conditions. The selected dyes are calcein (**a**), tetracycline (**b**) and alizarin red S (**c**) (top). Concentrations of Mn^2+^ (0, 0.25, 0.5, 0.75 and 1.0 mM on x-axes) vs. Mg^2+^ (4, 6, 8 mM, y-axes) and dye (right scale) are adjusted for a typical LAMP reaction. The SNR (fluorescence ratio between samples with 1.0 mM PPi and without) is represented by increasing colour from white to black (top). The comparison of selected SNR values (y-axes) from the screening (white) with the corresponding enzymatic assay (black) is presented in the lower part. The solid black line depicts SNR of 1. Measurements were carried out in triplicates.

**Figure 5:**
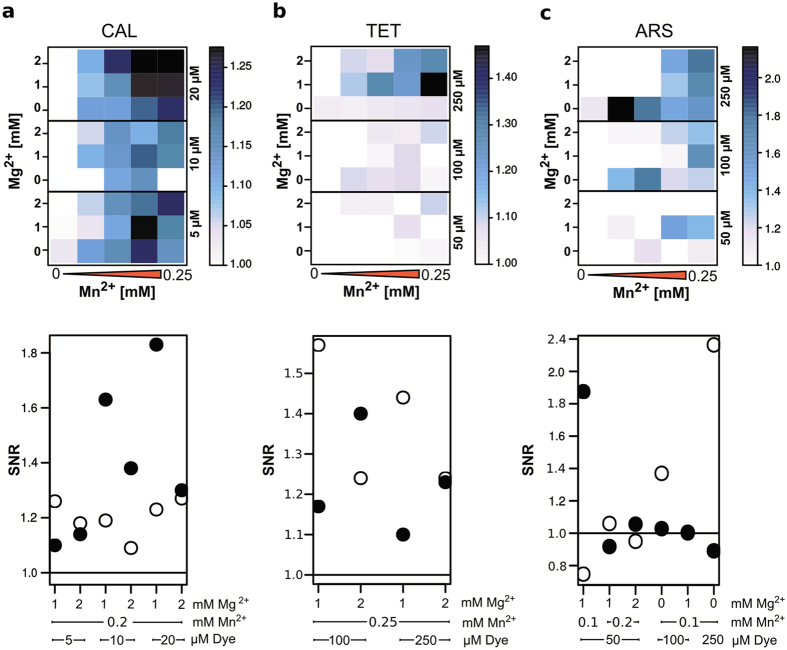
Heatmap analysis of signal-to-noise ratio (SNR) of simulated PCR conditions. The selected dyes are calcein (**a**), tetracycline (**b**) and alizarin red S (**c**). Mn^2+^ (0, 0.1, 0.15, 0.2 and 0.25 mM on x-axes), Mg^2+^ (0, 1, 2 mM y-axes) and dye (right scale) are adjusted for a typical PCR reaction. The SNR (fluorescence ratio between samples with 1.0 mM PPi and without) is represented by increasing colour from white to black (top). The comparison of selected SNR values (y-axes) from the screening (white) with the corresponding enzymatic assay (black) is presented in the lower part. The solid black line depicts SNR of 1. Measurements were carried out in triplicates.

**Figure 6:**
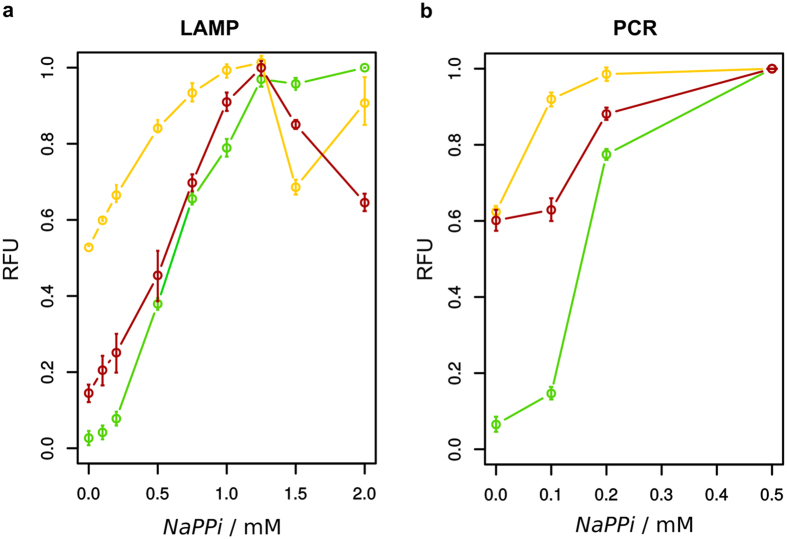
Fluorescence intensity measurements with CAL, TET and ARS in relation to increasing PPi concentration. Relative fluorescence of calcein (green), tetracycline (yellow) and alizarin red S (red) with increasing sodium pyrophosphate under reaction specific conditions (Supplemental Table 3) of LAMP (**a**) and PCR (**b**) was determined in a microtiter plate in 50 mM Tris/Hcl buffer (pH 8.8). Samples were prepared in triplicates.

**Figure 7:**
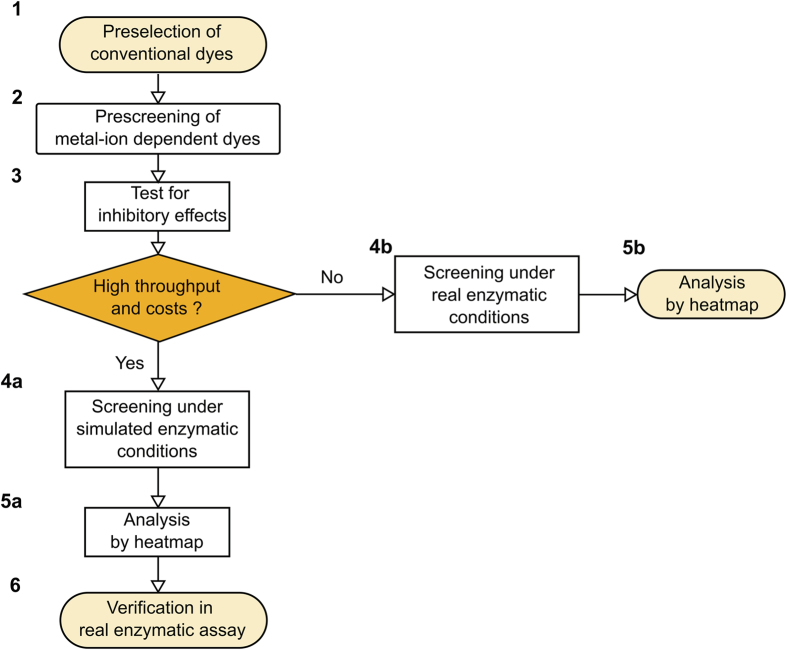
General screening workflow. Optimised workflow to determine the suitability of candidate dyes in fluorescence-based enzymatic assays based on displacement of metal ions by PPi.

The screening was carried out by fluorescence measurements in microtiter plate with each well having one combination of three dyes, three magnesium and five manganese ion concentrations (Supplementary Table 1) in presence and absence of 1.0 mM PPi. The microtiter plate layout (Fig. 1) depicts the plate configuration. Supplementary Table 2 summarizes the different combinations for each dye as well as the measurement parameters. The concentration range for Mn^2+^ in a LAMP reaction was adjusted to 0, 0.25, 0.5, 0.75 and 1.0 mM and in a PCR reaction to 0, 0.1, 0.15, 0.2 and 0.25 mM. Typical Mg^2+^ concentrations in LAMP are 4, 6 and 8 mM and in a PCR up to 2 mM. Fluorescence intensities were determined by a Cary Eclipse Fluorescence Spectrophotometer (Agilent Technologies, Santa Clara, USA) and selective excitation and emission wavelengths (Supplemental Table 1).

### Analysis of the combinatorial screening with heatmaps

The fluorescence intensities were used to calculate the signal-to-noise ratio (SNR) between samples with PPi (simulation) or DNA (real assay) and without PPi or DNA under identical conditions. The resulting value was used to generate a heatmap with R-statistical computing (www.r-project.org) and the “levelplot” package. The colorkey covers the range between the lowest (white) to highest (black) SNR value. The heatmap represents the SNR values by combining the used manganese, magnesium and dye concentrations in one image.

### Detection of inorganic pyrophosphate

The response of the fluorescent dyes to increasing sodium pyrophosphate (0 to 2 mM for LAMP, 0 to 0.5 mM for PCR) was investigated by fluorescence intensity measurements under specific LAMP and PCR reaction conditions (Supplemental Table 3) in a microtiter plate with a total volume of 100 μL/well in the plate reader Infinite^®^ 200 PRO (Tecan, Männedorf, Switzerland). The fluorescence values were plotted against the PPi concentration.

## Additional Information

**How to cite this article:** Fischbach, J. *et al*. Alizarin Red S for Online Pyrophosphate Detection Identified by a Rapid Screening Method. *Sci. Rep.* **7**, 45085; doi: 10.1038/srep45085 (2017).

**Publisher's note:** Springer Nature remains neutral with regard to jurisdictional claims in published maps and institutional affiliations.

## Supplementary Material

Supplementary Information
