# Supplementary material for: Alizarin Red S for Online Pyrophosphate Detection Identified by a Rapid Screening Method
Source: Sci Rep. 2017 Mar 24;7:45085. doi: 10.1038/srep45085 (PMC5364467; doi:10.1038/srep45085)
Supplement: Supplementary Information [file srep45085-s1.pdf]

Supporting Information

"Alizarin Red S for Online Pyrophosphate Detection Identified by a Rapid Screening Method"

Jens Fischbach , Qiuting Loh , Frank F. Bier, Theam Soon Lim , Marcus Frohme , Jörn Glökler

Table of Contents

1. Methods....................1
1.1 Selectivity of manganese to inorganic pyrophosphate and dNTPs....................1
1.2 Effect of manganese and magnesium on selected fluorescence dyes under LAMP and PCR conditions....................2
1.3 Effect of Mn^{2+}, tetracycline and alizarin red S on the Bst- and Taq DNA polymerase....................2
1.4 Spectral characterization of calcein, tetracycline and alizarin red S....................2
1.5 Combinatorial screening assay of calcein, calcein blue, folic acid, tetracycline and alizarin red S........3
1.6 LAMP and PCR assay....................3
2. Additional results with figures....................5
2.1 Selectivity of manganese to inorganic pyrophosphate (PPi) and dNTPs....................5
2.2 Effect of manganese and magnesium on selected fluorescence dyes under PCR conditions....................6
2.3 Signal-to-noise ratio of all tested dyes under LAMP and PCR conditions....................6
2.4 Spectral characterization of calcein, tetracycline and alizarin red S....................7
2.5 Combinatorial microtiter screening assay of folic acid, doxycycline and calcein blue under LAMP conditions....................8
3. References....................8

1. Methods

All chemicals were obtained from Sigma Aldrich, St. Louis, USA.

1.1 Selectivity of manganese to inorganic pyrophosphate and dNTPs

An initial experiment was performed to determine the selectivity of manganese. A system consisting of 25 µM calcein and 0.5 mM manganese(II) chloride was used to measure the fluorescence intensity under increasing concentration (0 , 0.6 and 1 mM) of total dNTPs (Thermo Fisher, Waltham, USA) or sodium pyrophosphate alone as well as the combination of both (additive). To further investigate the effect of Mg^{2+}, all samples were prepared with and without 6 mM magnesium(II) chloride. The resulting fluorescence was measured in a 0.2 mL reaction tube in the ESEquant Tube Scanner (FAM channel: 470/520 nm) (Qiagen, Hilden, Germany) at 25°C in a 50 mM Tris/Hcl buffer (pH 8.8) after an equilibration time of 20 minutes.

1.2 Effect of manganese and magnesium on selected fluorescence dyes under LAMP and PCR conditions

Calcein, calcein blue and folic acid were dissolved in 1 M NaOH first. To reach a final concentration of 2 mM solution, all dyes including tetracycline, doxycycline hydrochloride and alizarin red S were dissolved in

water. The pH was adjusted to 8.8 and all solutions were stored at 4°C in the dark until use. $MnCl_2$, $MgCl_2$ and sodium pyrophosphate were dissolved in water to a final concentration of 0.1 M. To demonstrate the competitive displacement of Mn^{2+} by pyrophosphate for calcein, tetracycline and alizarin red S , doxycycline, calcein blue and folic acid the following fluorescence intensities were determined in a 96 well microtiter plate (Corning® 96 well plates, clear bottom, Sigma Aldrich). Each well was supplied with 25 µM of each dye in 100 µL 1x Thermopol buffer (New England Biolabs, Frankfurt am Main, Germany). Sample one only contained 2 mM magnesium-chloride as reference. Sample two represents a combination of 7 mM magnesium(II) chloride and 0.5 mM manganese(II) chloride. Sample three contained a mixture of both ions and sample four included additional 1.4 mM sodium pyrophosphate to calculate the signal-to-noise ratio. The same measurements were conducted for PCR conditions including 25 µM dye, 2 mM magnesium(II) chloride, 0.1 mM manganese(II) chloride and 0.5 mM sodium pyrophosphate. The intensity measurement was conducted in a microtiter plate reader (Multiskan™ FC Microplate Photometer, Thermo Fisher, Waltham, USA) with excitation at 370 nm and emission at 520 nm for tetracycline and doxycycline, 535 nm and 645 nm for alizarin red S, 488 nm and 525 nm for calcein, 323 nm and 435 nm for calcein blue as well as 365 nm and 450 nm for folic acid. The signal-to-noise ratio was determined between the samples with and without pyrophosphate (PPi).

1.3 Effect of Mn^{2+}, tetracycline and alizarin red S on the Bst- and Taq DNA polymerase

The effects of Mn^{2+} (0, 0.1, 0.2, 0.5, 0.75 and 1 mM), tetracycline and alizarin red S (0, 0.05, 0.1, 0.2, 0.25 and 0.5 mM) were tested in a standard LAMP and PCR assay as disclosed below. The assays were carried out by testing increasing concentrations and analyzed by gel electrophoresis in a 2% agarose gel.

1.4 Spectral characterization of calcein, tetracycline and alizarin red S

Excitation and emission spectra of 0.1 mM dye in Tris/HCl-buffer (pH 8.8) were measured in the Infinite® 200 PRO plate reader (Tecan, Männedorf, Switzerland) in the range of 300 to 850 nm.

1.5 Combinatorial screening assay of calcein, calcein blue, folic acid, tetracycline and alizarin red S

Table 1. Concentration ranges of compounds in the combinatorial microtiter screening assay

Compound	[µM]	Excitation nm	Emission nm
**Calcein**	10, 25 and 50	495	525
**Tetracycline**	100, 250 and 500	370	520
**Folic acid**	25, 50 and 75	365	450
**Calcein blue**	10, 25 and 50	322	435
**Doxycycline**	100, 250 and 500	370	520
**Alizarin Red S**	50, 100 and 250	535	645
**Mn^{2+} ($MgCl_2$) (LAMP)**	0, 250, 500, 750 and 1000		
**Mn^{2+} ($MgCl_2$) (PCR)**	0, 100, 150, 200 and 250		
**Mg^{2+} ($MgSO_4$) (LAMP)**	4000, 6000, 8000		
**Mg^{2+} ($MgSO_4$) (PCR)**	1000, 2000		

1.6 LAMP and PCR assay

The initial experiments were carried out as simulations and selected results were verified by real enzymatic reactions including LAMP and PCR. To reduce the number of reactions, only combinations with the high signal-to-noise ratio in the simulation were selected. The LAMP-assay was based on the detection of potato spindle tuber viroid [1]. The PCR assay was conducted with the outer LAMP primer (F3 and B3, table 2). Dried PSTVd-positive material (PV0064) was purchased from the German strain culture collection (DSMZ, Braunschweig, Germany). The RNA extraction was done with the RNeasy Plant Mini Kit (Qiagen, Hilden, Germany). LAMP-Primers were designed according to Lenarčič *et al.* [2]. The isolated RNA was reverse transcribed into single-stranded DNA using the reverse primer and Maxima Reverse Transcriptase (Thermo Scientific, Schwerte, Germany). The DNA template for the LAMP reactions was generated by PCR using the protocol published by Weidemann *et al.* [3]. The 360 bp (PSTVd) products were purified with the MSB® Spin PCRapace Clean Up Kit (Stratec, Birkenfeld, Germany), quantified by Qubit® dsDNA HS Assay (Life Technologies, Darmstadt, Germany) and used directly in the assays. All oligonucleotides (Thermo Scientific) were HPLC purified.

Table 2. Overview of LAMP-primer for detection of PSTVd. * T_M is calculated with isothermal reaction conditions (1 µM of the Oligo, 20 mM monovalent ions, 8 mM divalent ions, 1 mM dNTPs).

**Locus: PSTVd (NC_002030.1)**		
**LAMP primer**	**primer sequence (5' – 3')**	**Tm * [°C]**
F3	AAAAAGGACGGTGGGGAG	63
B3	CCCCGAAGCAAGTAAGATAG	63
FIP (F1+F2)	GGAAGGACACCCGAAGAAAGG-GCCGACAGGAGTAATTCC	68 + 63
BIP (B1+B2)	GCTGTCGCTTCGGCTACTAC-AGAAAAAGCGGTTCTCGG	68 + 64
Lf	GGTGAAAACCCTGTTTCGG	64
Lr	CGGTGGAAACAACTGAAGC	64

The PSTVd-LAMP assay was set up in a total volume of 25 µL containing 1x supplied Thermopol reaction buffer including 2 mM $MgCl_2$, 1.2 mM of each dNTP (Thermo Scientific), 0.8 M betaine (Carl Roth, Karlsruhe, Germany), 0.32 U/µL Bst 3.0 DNA Polymerase (New England Biolabs), 1.6 µM of FIP and BIP primer, 0.4 µM of LF and LR pimer, 0.2 µM of F3 and B3 primer (Carl Roth) and 1 ng purified DNA template. Finally, dye, manganese and magnesium was added to the solution and adjusted to 50 µL with molecular grade water in a 0.2 mL reaction tube. The negative control was carried out without DNA template. The LAMP samples were incubated at 65 °C for 60 minutes in a thermocycler (Eppendorf AG, Hamburg, Germany). The PCR assay was set up in a total volume of 25 µL containing 1x Pol Buffer A (without $MgCl_2$), 1.25 U Taq DNA polymerase (Roboklon GmbH, Berlin, Germany), 0.2 mM each primer (F3 and B3), 0.25 mM each dNTP, 1 ng template DNA and dye, magnesium- and manganese-ions to reach the respective concentration. As for LAMP, the negative control was carried out without template DNA. The reaction tubes were incubated in a thermocycler at 95°C for 5 minutes, followed by 35 cycles of 50 sec. at 95°C, 30 sec. at 60°C and 30 sec. at 72°C. Each sample was transferred to a separate well in a microtiter plate. Dependent on the dye, the fluorescent intensity was determined by a microtiter plate reader at the specified wavelengths (Table 1).

2. Additional results with figures

2.1 Selectivity of manganese to inorganic pyrophosphate (PPi) and dNTPs

The selectivity of manganese for PPi is required to ensure that the signal response of the selected dyes in an enzymatic reaction (LAMP and PCR) is not significantly dependent on dNTPs. We used calcein as an established standard dye which is strongly quenched by manganese. The data indicate that in absence of Mg^{2+}, Mn^{2+} does not discriminate well between dNTPs and PPi. In Presence of excess magnesium, manganese is more selective to PPi than to the dNTPs. The additive effect of PPi with dNTPs revealed a signal increase that corresponds well to values of PPi and dNTP alone. Considering that dNTP is depleting as reactant and PPi is accumulating in a chemical reaction, Mn^{2+} has a higher affinity to PPi in presence of Mg^{2+}. Thus, all following experiments were performed without expensive dNTPs.

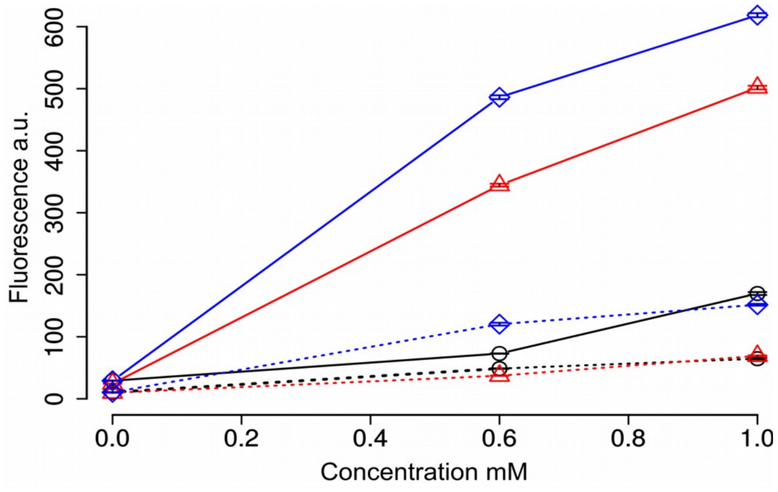

Figure S1. Selectivity of manganese to PPi and dNTPs using calcein/Mn^{2+} as reference. Fluorescence measurements were carried out with 25 μM calcein and 0.5 mM Mn^{2+} in presence (solid line) or absence (dashed line) of 6 mM Mg^{2+} in relation to increasing PPi (red) and dNTPs (black) as well as the combination (blue) in 50 mM Tris/Hcl (pH 8.8). The samples were prepared in triplicates and represented by error bars.

2.2 Effect of manganese and magnesium on selected fluorescence dyes under PCR conditions

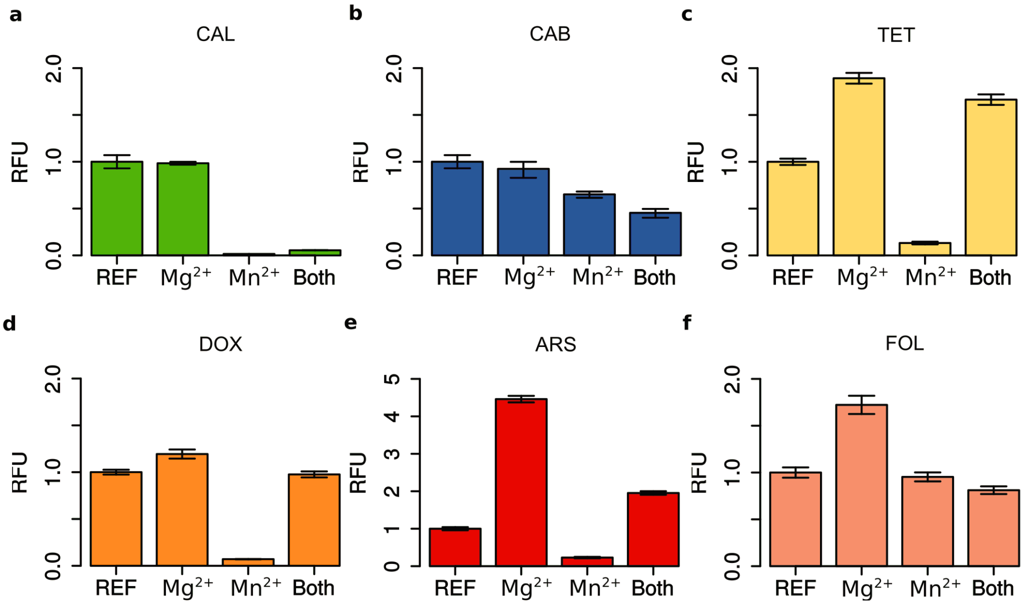

Figure S2. Prescreening of conventional fluorescence dyes with response to magnesium and/or manganese ions under PCR conditions. Fluorescence measurements were carried out with 25 µM calcein (a), calcein blue (b), tetracycline (c), doxycycline (d), alizarin red S (e) and folic acid (f) in 50 mM Tris/Hcl buffer (pH 8.8) with supplemented Mg^{2+} (2 mM, MG), Mn^{2+} (0.5 mM MN) as well as a combination of both. The values were normalized to the reference sample that represents the fluorescence of the dye alone. Values above 1.0 correlate with fluorescence increase and below with quenching. The samples were prepared in triplicates and represented by error bars.

2.3 Signal-to-noise ratio of all tested dyes under LAMP and PCR conditions

The signal-to-noise ratio (SNR) was calculated by measuring the responding fluorescence of samples with and without sodium pyrophosphate. The highest SNR was achieved with calcein, tetracycline and alizarin red S under LAMP conditions.

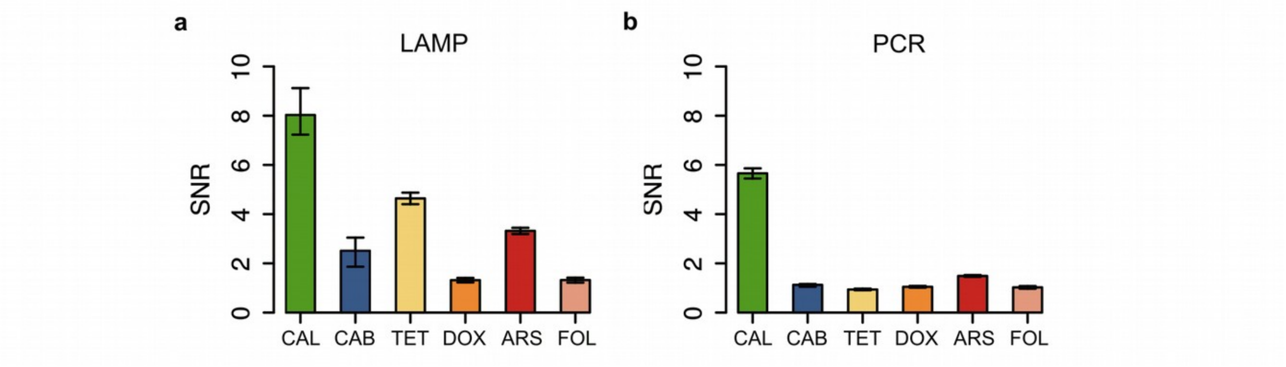

Figure S3. Signal response of fluorescence dyes in presence and absence of PPi under LAMP and PCR conditions. Signal-to-noise ratio of 25 µM mM calcein (CAL), calcein blue (CAB), tetracycline (TET), doxycycline (DOX), alizarin red S (ARS) and folic acid (FOL) between a sample containing 1.4 mM (LAMP) or 0.5 mM (PCR) sodium pyrophosphate and a corresponding sample without as reference. All LAMP samples contain 7 mM magnesium chloride and 0.5 mM manganese chloride (a). Samples for PCR contain 2 mM magnesium chloride and 0.5 mM manganese chloride (b). The samples were prepared in triplicates and represented by error bars.

2.4 Spectral characterization of calcein, tetracycline and alizarin red S

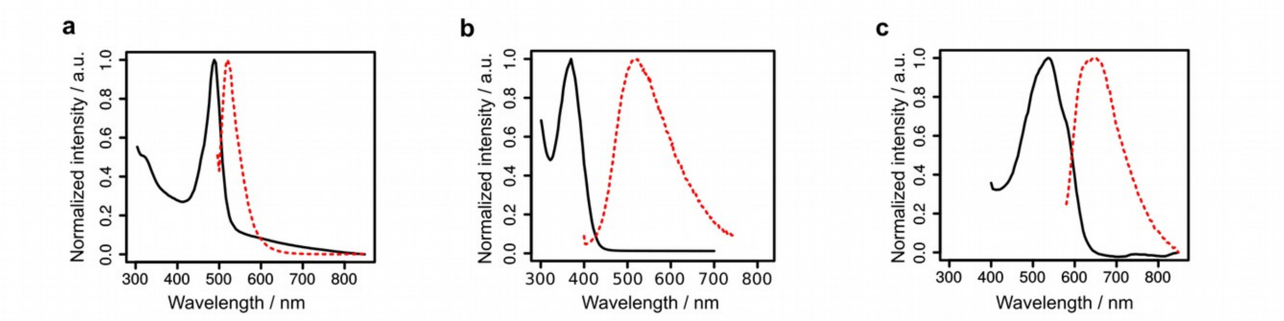

Figure S4. Characterization of the selected dyes. Excitation spectra (solid line) and emission spectra (dashed line) of calcein (a), tetracycline (b) and alizarin red S (c). The spectra were determined with 0.1 mM of each dye in 50 mM Tris/HCl (pH 8.8).

2.5 Combinatorial microtiter screening assay of folic acid, doxycycline and calcein blue under LAMP conditions

The investigated fluorescence dyes did result in poor SNR values and thus were omitted in subsequent experiments.

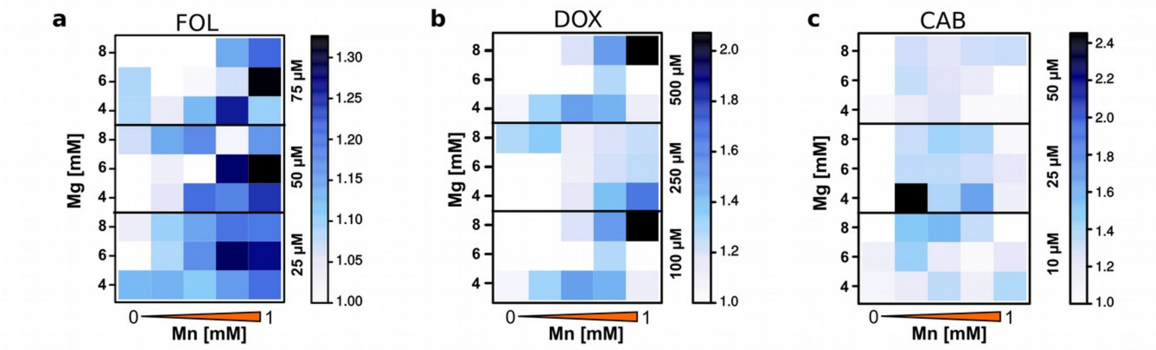

Figure S5.

Heatmap analysis of signal-to-noise ratio (SNR) of simulated LAMP conditions. The selected dyes are folic acid (a), Doxycycline (b) and Calcein blue (c). Concentrations of Mn^{2+} (0, 0.25, 0.5, 0.75 and 1.0 mM), vs. Mg^{2+} (4, 6, 8 mM) and dye (right scale). The SNR (fluorescence ratio between samples with 1.0 mM PPi and without) is represented by increasing color from white to black. Measurements were carried out in triplicates.

The combinatorial microtiter screening and the control by real reactions lead to following concentrations of magnesium, manganese and dye.

Table 3. Optimized dye, manganese and magnesium concentrations for sodium PPi dependent fluorescence measurements.

	**Calcein**		**Tetracycline**		**Alizarin red S**	
**Reaction**	LAMP	PCR	LAMP	PCR	LAMP	PCR
**Dye [mM]**	0.05	0.02	0.1	0.25	0.05	0.05
**Manganese [mM]**	0.5	0.2	0.75	0.2	0.75	0.1
**Magnesium [mM]**	8	1	4	1	4	1

3. References

1. Gross, H. J. Nucleotide sequence and secondary structure of potato spindle tuber viroid. *Nature*, **273**, 203-208 (1978).
2. Lenarčič, R., Morisset, D., Mehle, N., and Ravnikar, M. Fast real-time detection of Potato spindle tuber viroid by RT-LAMP. *Plant Pathol.*, **62**, 1147-1156 (2012).
3. Weidemann, H.-L. and Buchta,U. A simple and rapid method for the detection of potato spindle tuber viroid (PSTVd) by RT-PCR. *Potato Research* **41**, 1-8 (1998).
